# Letter from the Juvenile Protective Association

**Published:** 1908-07

**Authors:** L. G. Hardman, Clifford L. Anderson, William W. Landrum, W. G. Witham, E. C. Callaway, W. R. Hammond, Crawford Jackson, J. L. Sudleson

**Affiliations:** Executive Committee; Executive Committee; Executive Committee; Executive Committee; Executive Committee; Executive Committee; Executive Committee; Executive Committee


### CORRESPONDENCE

### LETTER FORM THE JUVENILE PROTECTIVE ASSOCIATION.

Dear Sir: For the sake of the children and for the good name of Georgia, let us remedy the evils which exist at the so-called State Reformatory. Please read the statements of a reliable eye-witness, Rev. D. W. Brannen, of Milledgeville: "There is one little building, a hundred feet long and fifty feet wide, two stories high, in which everything is done, cooking, eating and sleeping. The white boys and negroes sleep in the same building, on the same floor, in rooms adjacent. No education and training given, no school equipment whatever, and no real reformatory descipline tending to "fixed habits in morality, religion, and industry," and reformatory is really a Juvenile prison farm. I did not exaggerate. I suppressed.

*A Remedy.*—Why not authorize the white children to be removed from the Prison Farm to the Juvenile State Farm, with 426 acres of land in Jackson County, on the R. R. within six miles of Athens—a splendid location—and let the State make adequate provisions for wards of the State sent to this institution, also investigate annually how the money is spent for her wards, and how they are being trained.

*A Contrast.*—Relative to the work and purpose of the Juvenile State, pardon us for quoting to you the words of a wise and expert New Yorker: "Georgia is surely to be congratulated upon having grasped the highest ideal yet advanced for dealing with the juvenile delinquent, to develop citizenship through citizenship."

Our plan is to start with a $100,000.00 plant and ask the State to appropriate only one-fourth of this amount, on such conditions as the Legislature deems best.

For full information we are sending you under separate cover out 32 page illustrated booklet.

One word more, let negro juvenile criminals be sent to the

State Reformatory—or, we will take the negro delinquents and provided for them in *altogether separate quarters*, a mile apart.

*A Small Western State.*—Montana, *with one-fourth the population of Georgia*, and much less wealth, has a $200,000.00 plant for her reform school, and last year appropriated $42,000.00. How about Georgia?

Believing firmly that you wish to do the best for our most chanceless children, and also that we propose a real remedy for the difficulties referred to above, and further inviting any suggestion of change which you may think wise, we are,

Yours truly,

L. G. Hardman.
Clifford L. Anderson.
William W. Landrum.
W. G. Wi tham.
E. C. Callaway.
W. R. Hammond.
Crawford Jackson.
J. L. Sudleson.
Executive Committee

---

Dr. S. R. Roberts has recently published a pamphlet entitled, "The Battle of Sex, The White Life vs. The Red Light," in which he presents in a forcible manner urgent reasons for continence in single men. The subject of sex, puberty, marriage, masturbation, venereal diseases, the white life and "the battle," are discussed in a concise, systematic way and in language that can readily be understood by the laity.

The pamphlet, we believe, was prepared for college students, and undoubtedly a wide dissemmination of the facts brought out by Dr. Roberts will work great good in lessening the social evil.

---

A bill has been signed by the President providing for increase pay for officers and men of the Navy, which, according to the members of the Committee on Military Affairs, puts the officers of the Navy on a parity with those of the Army under the new army appropriation bill.

